# Employing bacterial microcompartment technology to engineer a shell-free enzyme-aggregate for enhanced 1,2-propanediol production in *Escherichia coli*

**DOI:** 10.1016/j.ymben.2016.02.007

**Published:** 2016-07

**Authors:** Matthew J. Lee, Ian R. Brown, Rokas Juodeikis, Stefanie Frank, Martin J. Warren

**Affiliations:** a School of Biosciences, University of Kent, Giles Lane, Canterbury, Kent CT2 7NJ, UK

**Keywords:** BMC, bacterial microcompartment, Pdu, 1,2-propanediol utilisation, 1,2-PD, 1,2-propanediol, D18, First 18 amino acids of PduD, P18, First 18 amino acids of PduP, Synthetic biology, Metabolic engineering, Compartmentalisation, Protein aggregation, Biotechnology

## Abstract

Bacterial microcompartments (BMCs) enhance the breakdown of metabolites such as 1,2-propanediol (1,2-PD) to propionic acid. The encapsulation of proteins within the BMC is mediated by the presence of targeting sequences. In an attempt to redesign the Pdu BMC into a 1,2-PD synthesising factory using glycerol as the starting material we added N-terminal targeting peptides to glycerol dehydrogenase, dihydroxyacetone kinase, methylglyoxal synthase and 1,2-propanediol oxidoreductase to allow their inclusion into an empty BMC. 1,2-PD producing strains containing the fused enzymes exhibit a 245% increase in product formation in comparison to un-tagged enzymes, irrespective of the presence of BMCs. Tagging of enzymes with targeting peptides results in the formation of dense protein aggregates within the cell that are shown by immuno-labelling to contain the vast majority of tagged proteins. It can therefore be concluded that these protein inclusions are metabolically active and facilitate the significant increase in product formation.

## Highlights

• Fusion of BMC targeting peptides to enzymes has a variable effect on activity.
• Tagged enzymes for 1,2-propanediol synthesis are localised to a BMC.
• BMC-targeted proteins localised within the BMC are protected from proteases.
• TEM reveals tagged enzymes form large intracellular protein aggregates.
• Strains with enzyme aggregates are shown to have enhanced 1,2-propanediol production.

## Graphical abstract

**Figure:**
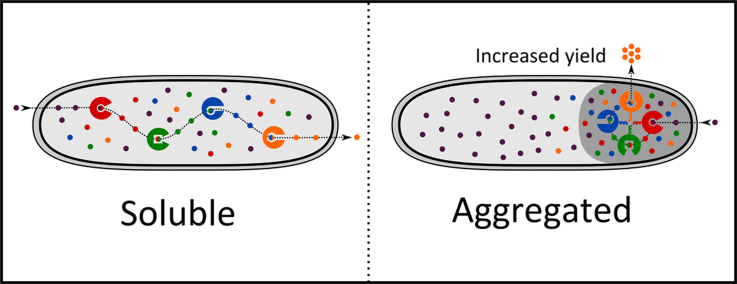

## 1 Introduction

Metabolic engineering involves the design and redesign of pathways and their deployment in organisms in which they do not naturally exist. This approach allows pathway fluxes, together with substrate and intermediate concentrations, to be manipulated by variation of the network parameters, which can be quantified by metabolic control analysis (Woolston et al., 2013). However, for pathways involving particularly volatile, unstable or toxic intermediates this tactic is likely to prove problematic. To overcome the problem of capricious metabolites nature has evolved a variety of solutions to ensure pathways operate efficiently without a significant build up of pernicious intermediates. In this respect substrate channelling, multienzyme complexes, metabolons and compartmentalisation are all ways in which pathway flux is naturally controlled (Lee et al., 2012).

In bacteria, compartmentalisation is mediated through the deployment of bacterial microcompartments (BMCs), which are used to address the problem of unstable or reactive intermediates (Dou et al., 2008, Havemann et al., 2002, Penrod and Roth, 2006, Sampson and Bobik, 2008). BMCs are proteinaceous complexes that are composed of a semi-porous capsid shell that encases a specific metabolic process (Cheng et al., 2008, Frank et al., 2013, Tanaka et al., 2008, Yeates et al., 2008). The widespread dispersal of BMCs in 23 bacterial phyla mediated through horizontal gene transfer suggests that pathway enhancement through employment of these structures provides a strong evolutionary benefit (Axen et al., 2014). There are two broad classes of BMCs, carboxysomes and metabolosomes, which are associated with either anabolic carbon fixation or catabolic carbon utilisation, respectively. Metabolosomes, in particular, appear to operate pathways that involve aldehydes as intermediates. Indeed, a recent bioinformatic analysis of BMC-associated operons revealed that the vast majority of these operons encode for aldehyde and alcohol dehydrogenases (Axen et al., 2014). The best characterised of the metabolosomes are those associated with 1,2-propanediol utilisation (Pdu) and ethanolamine utilisation (Eut), both of which house cobalamin-dependent enzymes and encase pathways that proceed via propanaldehyde and acetaldehyde respectively (Chowdhury et al., 2014). Compelling evidence has been presented that the compartments help protect the cell from toxicity associated with a high aldehyde concentration (Brinsmade et al., 2005, Sampson and Bobik, 2008, Cheng et al., 2011).

The ability to concentrate a specific metabolic pathway into what is essentially a nano-bioreactor, coupled with the capacity to sequester toxic pathway intermediates, has brought BMCs to the attention of synthetic biologists who view this as a tractable system that can be redesigned to accommodate new pathways (Chowdhury et al., 2014, Lawrence et al., 2014). Such a system has the potential to be used to enhance the yield of commodity chemicals produced via bacterial fermentation. Significantly, the *Citrobacter freundii* Pdu BMC can be produced as an empty compartment through the coordinated production of only the shell proteins (PduA, B, B′, J, K, N, U) (Parsons et al., 2010). Enzymes can then be targeted so that they are incorporated into the BMC through the fusion of peptide sequences that are found at the N-terminus of proteins such as PduD (D18) and PduP (P18) (Fan et al., 2010; Fan and Bobik, 2011). Furthermore, targeting of the *Zymomonas mobilis* pyruvate decarboxylase and alcohol dehydrogenase resulted in the conversion of the Pdu BMC into an ethanol bioreactor (Lawrence et al., 2014).

Bio-product commodities that have successfully transitioned into the market through biotechnological approaches include 1,3-propanediol, polylactic acid (PLA) and polyhydroxyalkanoate (PHA), which have been used for personal care products, antifreeze and biodegradable plastics (Adkesson et al., 2011, Jung et al., 2010, Suriyamongkol et al., 2007). Near-term bio-based products, such as 1,4-butanediol, isobutanol and succinic acid are in progress whilst systems are under development for the production of terpenes and itaconic acid (Burk et al., 2011, Lee et al., 2005, Peralta-Yahyan et al., 2011, Peralta-Yahya et al., 2012, Steiger et al., 2013, Yim et al., 2011). Approaches such as metabolic engineering and synthetic biology are routinely applied in order to make these processes more efficient and cost competitive. In this paper we outline a method that offers the potential for a significant step-change in bio-commodity production through the development of BMC technology.

**Fig. 1:**
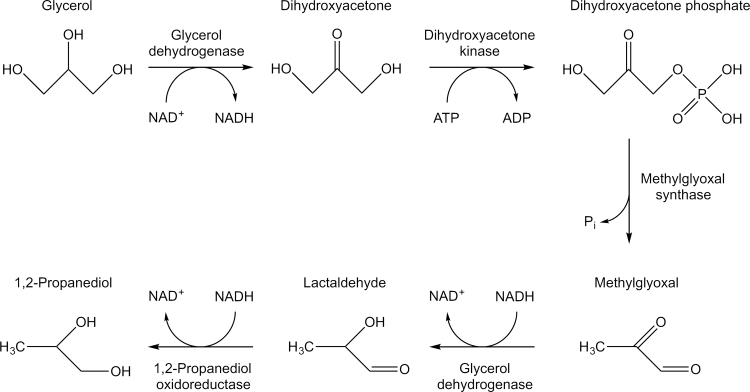
A pathway for the synthesis of 1,2-propanediol from glycerol. Glycerol dehydrogenase and dihydroxyacetone kinase catalyse the conversion of glycerol to dihydroxyacetone phosphate. Methylglyoxal synthase catalyses the conversion to methylglyoxal. Glycerol dehydrogenase and 1,2-propanediol oxidoreductase catalyse the conversion of methylglyoxal to 1,2-propanediol via the intermediate lactaldehyde.

The production of 1,2-propanediol from glycerol is similarly recognised as a commercially relevant pathway. 1,2-Propanediol is a commodity chemical that is currently used in the production of plasticisers, antifreeze, thermoset plastics and cosmetics with an annual global demand estimated at around 1.36 million tonnes per year with demand expected to increase over the next few years (Clomburg and Gonzalez, 2011). It is, therefore, of great interest to develop a production method that does not rely on a non-renewable resource (Altaras and Cameron, 1999, Altaras and Cameron, 2000, Clomburg and Gonzalez, 2011). Glycerol, on the other hand, is readily available as it is produced as a by-product of the biodiesel production process (Marchetti et al., 2007). It has been reported that for every 100 kg of biodiesel produced 10 kg of glycerol is generated (Yazdani and Gonzalez, 2007). The biochemical pathway for the synthesis of 1,2-propanediol from glycerol (Fig. 1) involves the intermediate methylglyoxal, a compound that is highly toxic to cells in sub-millimolar concentrations (Ferguson et al., 1996). The pathway involves four enzymes, glycerol dehydrogenase (GldA), dihydroxyacetone kinase (DhaK), methylglyoxal synthase (MgsA) and 1,2-propanediol oxidoreductase (FucO). Previously it has been shown that a DNA scaffold enhances 1,2-propanediol production in *Escherichia coli* from glucose with 3 enzymes including MgsA and GldA (Conrado et al., 2012). Here we set out an alternative approach to determine if the proposed pathway for 1,2-propanediol production could be enhanced through compartmentalisation into a BMC. Moreover we present an alternative approach to compartmentalisation that is the aggregation of enzymes into a supramolecular conglomerate.

The aim of the investigation was therefore to set about creating fusion proteins between known Pdu targeting peptides (D18 and P18) and the four 1,2-propanediol producing enzymes in order to allow their targeting to a recombinant empty Pdu BMC system. The effect of the targeting peptides on the activity of the different enzymes and their solubility was investigated. The ability of the targeted enzymes to promote 1,2-propanediol synthesis was determined. The strains were analysed by TEM and protein aggregation was found to play an unexpected but key role in enhancing pathway productivity.

## 2 Materials and methods

### 2.1 Plasmid construction

Plasmids were constructed to provide each of the genes of interest with a N-terminal hexa-histidine tag with an optional D18 or P18 targeting peptide.

All primers used in this study are listed in Supplementary Table 3. All genes were amplified with flanking *Nde*I and *Spe*I restriction sites and each was ligated into pET14b, pET14b-D18 and pET14b-P18 vectors using *Nde*I and *Spe*I restriction sites. Plasmids pML-1 to pML-6 as outlined in Supplementary Table 2, were constructed by a ‘Link and Lock’ approach utilising the compatible sticky ends formed by digestion with *Xba*I and *Spe*I (McGoldrick et al., 2005).

### 2.2 Overexpression and purification of recombinant protein

BL21 (DE3) pLysS competent cells were transformed with a plasmid containing the gene(s) of interest. 1 L of LB supplemented with ampicillin (100 mg/L) and chloramphenicol (34 mg/L) in baffled flasks was inoculated from an overnight starter culture. The cultures were grown at 37 °C with shaking for 7 h; protein production was induced by the addition of IPTG to a final concentration of 400 µM. The cultures were then incubated overnight at 19 °C with shaking. Cells were harvested by centrifugation at 3320×*g* for 15 minutes at 4 °C, pellets were resuspended in 20 mM Tris–HCl pH 8.0, 500 mM NaCl, 5 mM imidazole. Cells were lysed by sonication and cell debris removed by centrifugation. Recombinant protein was then purified from the soluble fraction by immobilized metal ion affinity chromatography.

### 2.3 Activity assays

#### 2.3.1 Glycerol dehydrogenase

The activity of GldA for the oxidation of glycerol to dihydroxyacetone was measured by following the initial rate of reduction of NAD^+^ to NADH at 340 nm. Activity assays were carried out in 1 mL reactions containing 0.1 M potassium phosphate buffer pH 8.0, 500 μM NAD^+^, 2 mM MgCl_2_ and 200 nM GldA. The activity of GldA for the reduction methylglyoxal to lactaldehyde, was measured by following the initial rate of the oxidation of NADH to NAD^+^ at 340 nm. Activity assays were carried out in 1 mL reaction containing 0.1 M potassium phosphate pH 8.0, 0.1 mM NADH, 2 mM MgCl_2_ and 200 nM GldA.

#### 2.3.2 Dihydroxyacetone kinase

The activity of DhaK for the conversion of dihydroxyacetone to dihydroxyacetone phosphate was measured in a coupled reaction with Glyceraldehyde 3-phosphate dehydrogenase (G3PDH) by following the oxidation of NADH to NAD^+^ at 340 nm. Activity assays were carried out in 1 mL reactions containing 50 mM Tris–HCl pH 7.5, 100 mM NaCl, 1 mM ATP, 0.1 mM NADH, 2.5 mM MgCl_2_, 7.2 U G3PDH and 125 nM DhaK.

#### 2.3.3 Methylglyoxal synthase

The activity of MgsA was monitored in a colorimetric assay over a time course. 25 μL of 0.5 mM MgsA was incubated in a reaction mixture containing 400 μL 50 mM imidazole pH 7.0, 25 μL 15 mM dihydroxyacetone phosphate and 50 μL *d*H_2_O, the reaction mixture was incubated at 30 °C with shaking. At time intervals 50 μL of the reaction mixture was removed and added to a detection mixture containing 450 μL dH_2_O, 165 μL 0.1% 2,4-dinitrophenylhydrazine hydrochloric acid solution. The detection mixture was incubated at 30 °C with shaking for 15 min. 835 μL of 10% (w/v) NaOH was added to the detection mixture which was incubated at room temperature for 15 minutes. Absorbance was then measured at 550 nm.

#### 2.3.4 1,2-Propanediol oxidoreductase

The activity of FucO was determined for the NADH dependant reduction of glycolaldehyde to elthylene glycol was measured by following the initial rate of the oxidation of NADH to NAD^+^ at 340 nm. Activity assays were carried out in 1 mL reactions containing 100 mM Hepes pH 7.5, 10 μM NADH, 100 μM MnCl_2_ and 200 nM FucO.

### 2.4 Culture medium and conditions for 1,2-propanediol production

The culture medium designed by Neidhardt et al. (1974) was supplemented with 30 g/L glycerol, 10 g/L tryptone and 5 g/L yeast extract. Strains were cultured in sealed serum bottles with a working volume of 100 mL at 28 °C with shaking. Cultures were inoculated from starter cultures to a starting OD_600_ of 0.05. During growth 1 mL samples were removed at 0, 6, 12, 24, 48, 72 and 96 h for analysis of 1,2-propanediol content.

### 2.5 Western blot analysis

Nitrocellulose membranes following transfer and blocking were incubated in primary antibody (mouse anti-His (Sigma Aldrich) 1:3000 or mouse anti-GFP (Sigma Aldrich) 1:1000) followed by incubation in a secondary coupled antibody coupled to alkaline phosphatase (Anti-Mouse IgG (H+L), AP Conjugate (Promega) 1:5000). Bands were visualised by incubation in substrate 5-bromo-4-chloro-3-indolyl phosphate/nitro blue tetrazolium (BCIP/NBT).

### 2.6 Analysis of 1,2-propanediol production

In-vivo 1,2-propanediol production was determined by GC/MS analysis of the growth medium at time intervals (0, 6, 12, 24, 48, 72 and 96 h). The supernatant after centrifugation, was boiled for 10 min at 100 °C followed by centrifugation at 19,750×*g*. The sample was then acidified with trifluoroacetic acid to a final concentration of 0.01% followed by a second centrifugation at 19,750×*g*. The supernatant following centrifugation was diluted 1:4 in acetonitrile for GC/MS analysis.

### 2.7 Visualisation of engineered strains

#### 2.7.1 Embedding of strains for TEM analysis

Strains were embedded, sectioned and stained as described in supplementary information.

#### 2.7.2 Embedding of strains for immunolabelling

Strains were cultured as described previously (Section 2.4) overnight, cells were harvested by centrifugation for 10 min at 3000×*g*. The cell pellet was resuspended in 2% formaldehyde and 0.5% gluteraldehyde in 100 mM sodium cacodolate pH 7.2 and incubated for 2 h with gentle rotating. Cells were pelleted by centrifugation at 6000×*g* for 2 min and were washed twice for 10 min with 100 mM sodium cacodylate pH 7.2. This was followed by dehydration of the samples in an ethanol gradient, 50% EtOH for 10 min, 70% EtOH for 10 min, 90% EtOH for 10 min, followed by three 15 min washes in 100% EtOH. Cell pellets were then resuspended in 2 mL LR white resin and incubated overnight with rotation at room temperature after which the resin was changed and incubated for a further 6 h. Cell pellets were resuspended in fresh resin and transferred to 1 mL gelatine capsules and centrifuged at 4000×*g* to pellet the cells at the tip. Samples were polymerised at 60 °C for 24 h. Samples were ultra-thin sectioned on a RMC MT-XL ultramicrotome with a diamond knife (diatome 45°) sections (60–70 nm thick) were collected on 300 mesh gold grids.

#### 2.7.3 Immunolabelling of sections

Grids were equilibrated in one drop of TBST (20 mM Tris–HCl pH 7.2, 500 mM NaCl, 0.05% Tween 20, 0.1% BSA) before being transferred into a drop of 2% BSA in TBST and incubated at room temperature for 30 min. Grids were then immediately transferred into a 20 μL drop of primary antibody (mouse anti-his (Sigma Aldrich 1:10)) and incubated for 1 h. Grids were washed in a fresh drop of TBST followed by washing for 10 s in a stream of TBST. Grids were equilibrated in a drop of secondary antibody (Goat anti-mouse IgG 10 nm gold (Agar Scientific 1:50)) then incubated for 30 min in a fresh drop. Excess antibody was removed by washing in two drops of TBST before washing in a stream of ddH_2_O and dried.

#### 2.7.4 Staining of immunolabelled sections

Grids were stained for 15 minutes in 4.5% uranyl acetate in 1% acetic acid solution followed by 2 washes in dH_2_O. Grids were then stained with Reynolds lead citrate for 3 min followed by a wash in ddH_2_O. Electron microscopy was performed using a JEOL-1230 transmission electron microscope equipped with a Gatan multiscan digital camera at an accelerating voltage of 80 kV.

## 3 Results

### 3.1 Effect of targeting peptides on the activities of GldA, DhaK, MgsA and FucO.

Recently, it has been shown that fusing the targeting peptides D18 and P18 to heterologous enzymes, such as the *Z. mobilis* pyruvate decarboxylase and alcohol dehydrogenase, allowed their targeting to recombinantly produced empty BMCs, resulting in the formation of a functional ethanol bioreactor (Lawrence et al., 2014). However, the general effect of such fusions on functionality of individual enzymes had not been investigated in detail. In this study the enzymes involved in the microbial synthesis of 1,2-propanediol from glycerol, namely glycerol dehydrogenase (GldA), dihydroxyacetone kinase (DhaK), methylglyoxal synthase (MgsA) and 1,2-propanediol oxidoreductase (FucO) were cloned separately with both the D18 and P18 N-terminal targeting peptides followed by a hexa-histidine tag. The resulting proteins were purified by IMAC and the kinetic parameters of each of the protein fusions were subsequently determined and compared to enzymes containing only the N-terminal hexa-histidine tag. Herein, we refer to the D18-His and P18-His containing proteins as ‘tagged’ enzymes and the His-only containing proteins as ‘untagged’.

**Fig. 2:**
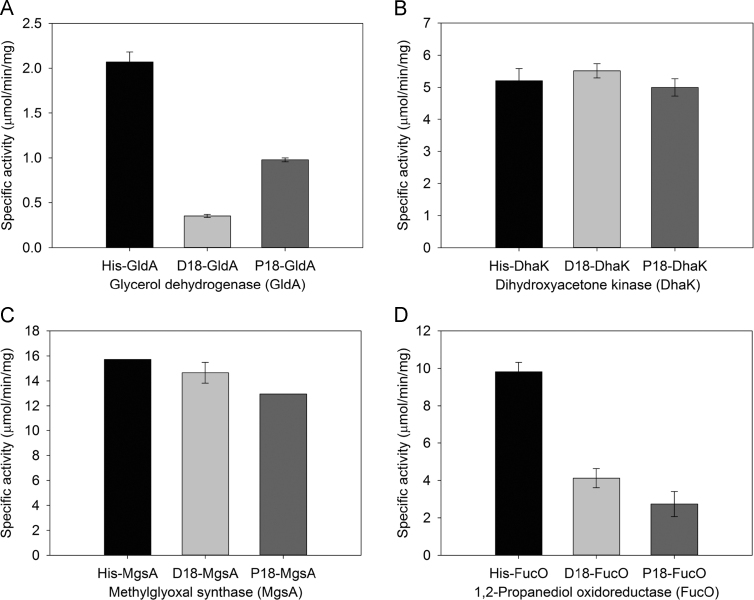
The effect of targeting peptides on the specific activities of the enzymes involved in the microbial synthesis of 1,2-propanediol. (a) glycerol dehydrogenase (for the reduction of methylglyoxal) (b) dihydroxyacetone kinase (c) methylglyoxal synthase (d) 1,2-propanediol oxidoreductase.

The targeting peptides were found to have a highly variable effect on the specific activities of the enzymes (Fig. 2). The first of the enzymes, GldA, is involved in two distinct steps in the transformation of glycerol to 1,2-propanediol, being responsible for the dehydrogenation of glycerol to dihydroxyacetone as well as the reduction of methylglyoxal to lactaldehyde. With respect to the activity of GldA in the dehydrogenation of glycerol, the D18 targeting peptide resulted in a decrease of 90% in the enzyme׳s specific activity in comparison to the un-tagged control. Tagging GldA with the P18 targeting peptide had a less dramatic effect although the specific activity was still reduced by 55%. The tags had a similar effect on the activity of GldA to catalyse the reduction of methylglyoxal to lactaldehyde, with the presence of D18 decreasing the specific activity by 83% whilst P18 reduced activity by 53% (Fig. 2a). In contrast, the activity of DhaK, which catalyses the ATP dependent phosphorylation of dihydroxyacetone, was not significantly affected by the presence of either targeting peptide (Fig. 2b). Fusing MgsA, the methylglyoxal synthase, with either the D18 or P18 targeting peptide had only a slight detrimental effect on enzyme activity, decreasing the activity by 15% and 18% respectively (Fig. 2c). The activity of FucO, the 1,2-propanediol oxidoreductase, was found to decrease by 58% when fused to D18 but was more significantly affected by the fusion of P18, which resulted in a 76% decrease in specific activity (Fig. 2d). It is, therefore, interesting to note that although the D18 targeting peptide is predicted to be structurally similar to P18, the two tags were found to have a differential influence on the activities of the same enzymes. From the data presented in Fig. 2 the most active forms of the enzymes to be taken forward for inclusion into a BMC were identified as P18-GldA, P18-DhaK, D18-MgsA and D18-FucO.

### 3.2 Targeting peptides cause a degree of protein aggregation

The production levels and solubility of GldA, DhaK, MgsA and FucO, with and without targeting peptides, were investigated by comparing samples obtained during their purification by denaturing polyacrylamide gel electrophoresis (Supplementary Figs. S1–S4). DhaK and MgsA were both found to be well produced and soluble irrespective of the presence of the D18 or P18 targeting peptide. The solubility of FucO was, similarly, not affected by the presence of the targeting peptides, although the yield of un-tagged FucO appeared to be slightly lower. In contrast, even though both P18 and D18-tagged GldA appeared to be produced and could be purified, more of the protein was detected in the insoluble fractions (Supplementary Fig. S1, lane 3). This suggests that the fusion proteins D18-GldA and P18-GldA had a greater tendency to aggregate in comparison to untagged GldA. Moreover, P18-GldA was also found to elute from the IMAC column with an additional band of lower molecular mass, indicative of partial protein degradation.

**Fig. 3:**
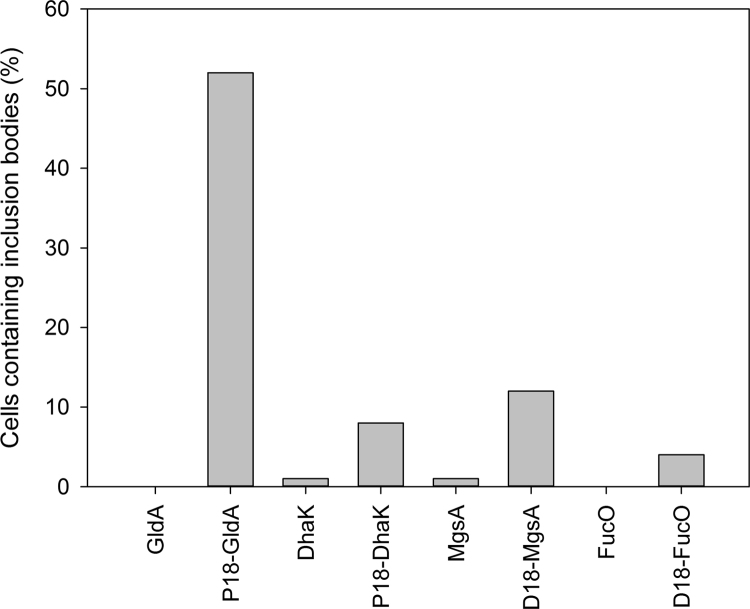
Statistical analysis showing the percentage of cells expressing tagged/untagged proteins containing inclusion bodies. For each strain 100 cells were observed by TEM.

Previous research has shown that the solubility of some of the enzymes of both the Pdu and Eut metabolosomes is increased by the removal of the N-terminal targeting peptides (Fan and Bobik, 2011; Shibata et al., 2010). Therefore the N-terminal peptides are known to affect solubility. It is clear that the addition of both D18 and P18 to GldA results in a decrease in solubility, most likely through protein aggregation. In order to investigate the aggregation behaviour of the tagged proteins in vivo, the most active protein fusions (P18-GldA, P18-DhaK, D18-MgsA and D18-FucO) were selected for TEM analysis of sections through whole cells. In this respect strains encoding each of the tagged proteins were cultured overnight without induction. Subsequently, the cells were harvested, embedded in low viscosity resin, thin sectioned and visualised using TEM. These were then compared to control strains that produced the untagged protein. For each strain 100 cells were examined and the statistical analysis of each of the strains is shown in Fig. 3. Representative TEM micrographs were compiled and are shown in Supplementary Fig. S5.

Control strains producing un-tagged proteins (GldA, DhaK, MgsA, FucO) displayed a ‘normal’ phenotype, with only a maximum of 1% of the observed cells containing electron dense areas that could be considered indicative of aggregated proteins. In contrast, half of all observed cells (52%) producing P18-GldA showed protein aggregates, which were located mainly at the poles of the cell (Supplementary Fig. S5 B). The addition of the P18 targeting peptide to the N-terminus of DhaK resulted in aggregate formation in 8% of the observed cells. Fusion of the D18 targeting peptide to MgsA and FucO resulted in the presence of protein aggregates in 12% and 4% of cells respectively. These results confirm that the fusions between the enzymes of the 1,2-propanediol production pathway and the D18 and P18 targeting peptides cause protein aggregation to various extents.

### 3.3 Proteins with targeting peptides are recruited to BMCs

The D18 and P18 fusion proteins were investigated for their ability to be targeted to an empty recombinant BMC. This was achieved by generating strains with the ability to co-express, individually, *P18-gldA*, *D18-dhaK, P18-mgsA* and *D18-fucO* together with the construct housing the genes for empty shell formation (pLysS-*pduABJKNU*). After growth, the recombinant BMCs were purified using a combination of centrifugation and differential salt precipitation as described previously (Lawrence et al., 2014). The isolated BMCs were then analysed by SDS PAGE for the presence of the characteristic BMC shell–protein profile. This revealed that all the tagged proteins co-purified with the BMC fraction to a greater extent than untagged protein, consistent with the proteins being encased within the BMC (Supplementary Fig. S6). This was further confirmed by kinetic assays of the final purified BMC fraction for the respective tagged enzymes.

**Fig. 4:**
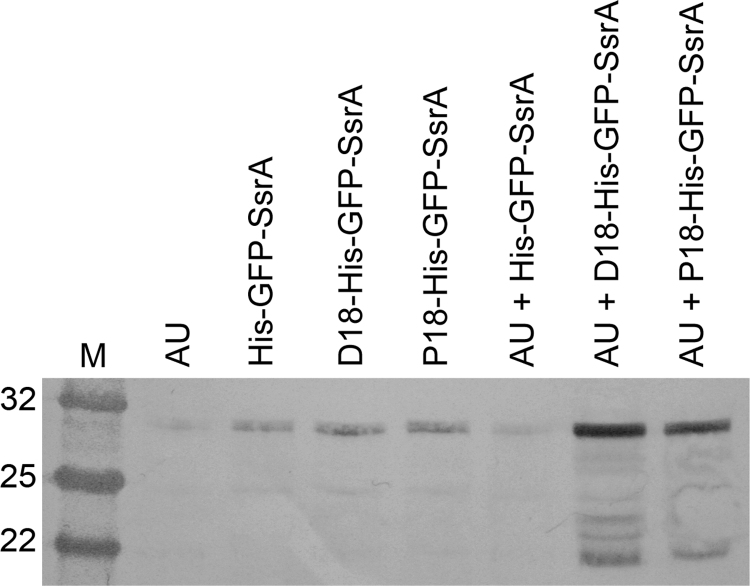
Protease protection assay of GFP fused to a C-terminal proteolysis tag and an N-terminal targeting peptide in the presence and absence of BMCs. Total lysates were analysed by SDS-PAGE and subsequently western blotted with an anti-GFP primary antibody. Cell densities were normalised to an OD_600_=2.5 for loading of samples. The faint bands seen in lanes 2-6 may, in part, be a result of unspecific binding, as a faint band is also seen in lane 1 (shell proteins only, no GFP).

To provide further evidence that the D18 and P18 fusions result in BMC encasement we chose to use a protease protection assay that was previously reported by Sargent et al. (2013). In this assay we used GFP as a marker to demonstrate that the protein is protected within the confines of the BMC. To this end plasmids were constructed containing GFP fused to an N-terminal D18 or P18 tag as well as a C-terminal SsrA proteolysis tag (AANDENYALAA*). The C-terminal SsrA tag targets proteins for degradation by the *E. coli* proteases ClpAP and ClpXP (Farrell et al., 2005). *E. coli* was transformed with plasmids encoding the protein fusions with and without shell proteins and the resulting strains were cultured for 24 h. Samples were taken and analysed by SDS-PAGE and subject to western blotting using an anti-GFP primary antibody. The results show that the co-expression of GFP-SsrA fused to targeting peptides, when produced with shell proteins, have the highest levels of GFP (Fig. 4; lanes 7+8). In the absence of a targeting peptide GFP is effectively degraded as observed by the presence of only a faint band present (Fig. 4; lane 6). In the absence of shell proteins all GFP fusion proteins are present to a much lesser extent than fusion proteins in the presence of microcompartments. These results are consistent with the theory that BMCs provide protection for internalised proteins.

### 3.4 Construction of 1,2-propanediol producing strains

The untagged genes for the four pathway enzymes were cloned consecutively using the Link and Lock procedure to give pML5 (containing *gldA, mgsA, dhaK, fucO*) whereas the tagged versions were cloned in a similar fashion to give pML6 (containing *P18-gldA, D18-mgsA, P18-dhaK, D18-fucO*). Both plasmids were transformed into *E. coli* strain BL21*(DE3). The strains were further engineered to co-express the 1,2-propanediol production plasmids by transforming them with a compatible plasmid housing the shell protein genes (pLysS-*pduABJKNU*). Additionally, a control strain containing empty versions of pET14b and pLysS was generated as was a strain transformed with pET14b and pLysS-*pduABJKNU*. All strains were compared for the production of 1,2-propanediol.

The strains were grown in 100 mL cultures at 28 °C and samples were collected at 0, 6, 12, 24, 48, 72 and 96 h. The resulting growth curves (Supplementary Fig. S7) show that strains encoding proteins with targeting peptides (either in the presence of absence of shell proteins) grow slower and reach a lower final optical density in comparison to strains expressing un-tagged proteins and control strains. When analysing the protein profiles of whole cell samples at the various time points we found marginally higher production levels of some tagged proteins compared to the non-tagged proteins (Supplementary Fig. S8 c, d, e and f). Increased protein production and protein aggregation may lead to cells undergoing senescence mediated by asymmetric segregation of protein aggregates (Baig et al., 2014), which potentially explains why these strains did not grow as well as strains producing the same proteins without targeting peptides. Whole cell samples of strains housing the shell protein construct (pLysS-*pduABJKNU*) showed the protein profile expected for the shell components as observed by SDS-PAGE (Supplementary Fig. S8 b, e and f). The strain encoding only empty microcompartments appears to have a slightly higher production of shell proteins as evidenced by increased band intensity on the SDS-PAGE gels (Supplementary Fig. S8 b).

### 3.5 In vivo 1,2-propanediol production is elevated in strains producing enzymes with targeting sequences

**Fig. 5:**
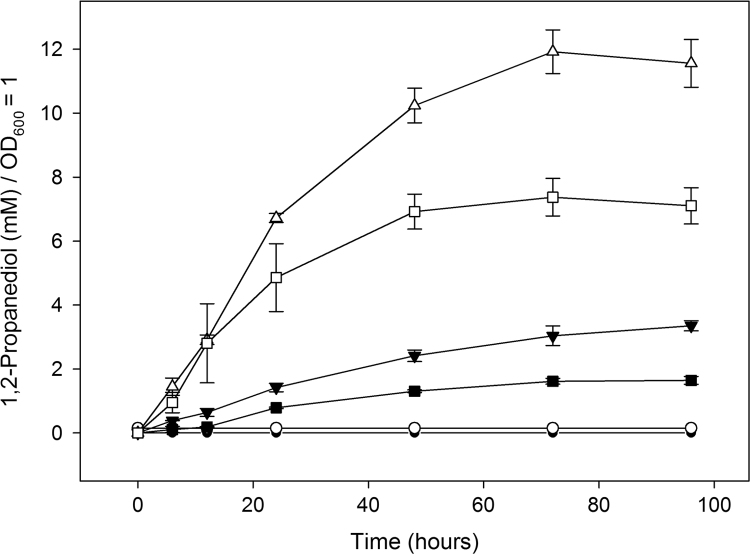
In vivo 1,2-propanediol production. The graph shows the 1,2-propanediol content (normalised to OD_600_=1) over 96 h in the growth medium of strains that lack shell proteins and 1,2-propanediol producing enzymes (control strain) (●), shell proteins only (control strain) (◯), untagged 1,2-propanediol producing enzymes (▼), 1,2-propanediol producing enzymes tagged with targeting peptides (△),untagged 1,2-propanediol producing enzymes and shell proteins (■),1,2-propanediol producing enzymes tagged with targeting peptides and shell proteins (⎕). Data points represent an average of three independent experiments; standard deviations are represented by error bars.

The 1,2-propanediol content in the growth media of the various strains was quantified by GC–MS. Samples were collected at 0, 6, 12, 24, 48, 72 and 96 h and, following centrifugation, the resultant supernatant was analysed by GC–MS as described in materials and methods. The measured 1,2-propanediol content, as shown in Fig. 5, is expressed for a cell density of OD_600_=1 (for the non-adjusted data see Supplementary Fig. S9). Strains encoding the un-tagged enzymes, His-GldA, His-DhaK, His-MgsA and His-FucO*,* whether in the presence of absence of BMCs, were found to produce low levels of 1,2-propanediol despite growing well and reaching the highest cell densities after 96 h.

Intriguingly, the highest levels of 1,2-propanediol were detected in the growth media of strains producing enzymes containing the fused targeting peptides. Thus strains producing P18-GldA, P18-DhaK, D18-MgsA and D18-FucO, whether in the presence or absence of BMCs, grew to a lower cell density than the strains harbouring un-tagged proteins, and despite the negative effect the targeting peptide has on the specific activities of the individual enzymes they produced significantly more 1,2-propanediol (Fig. 2, Fig. 5, and Supplementary Fig. S9) than the strains lacking the targeting peptides. The highest final yield of 1,2-propanediol was 11.56 mM/OD_600_ unit, which was observed when the shell proteins were not present. No 1,2-propanediol was detected in control stains (wild type *E. coli* and a strain producing shell proteins only).

### 3.6 TEM analysis of 1,2-propanediol producing strains

The higher product yield exhibited by the strain producing tagged enzymes in the absence of shell proteins was unexpected. To investigate if aggregation of the enzymes was causing this effect electron microscopy and immuno-gold labelling were used to visualise the subcellular organisation and location of the recombinant proteins in the various strains after the cells had been thin sectioned. To achieve this the thin sections of the strains were labelled with anti-histidine primary antibody, which is designed to bind to the hexa-histidine tag on the N-terminus of the various proteins. A secondary antibody conjugated to 10 nm gold particles was used to bind to the primary antibody, thereby revealing the intracellular location of 1,2-propanediol producing enzymes.

**Fig. 6:**
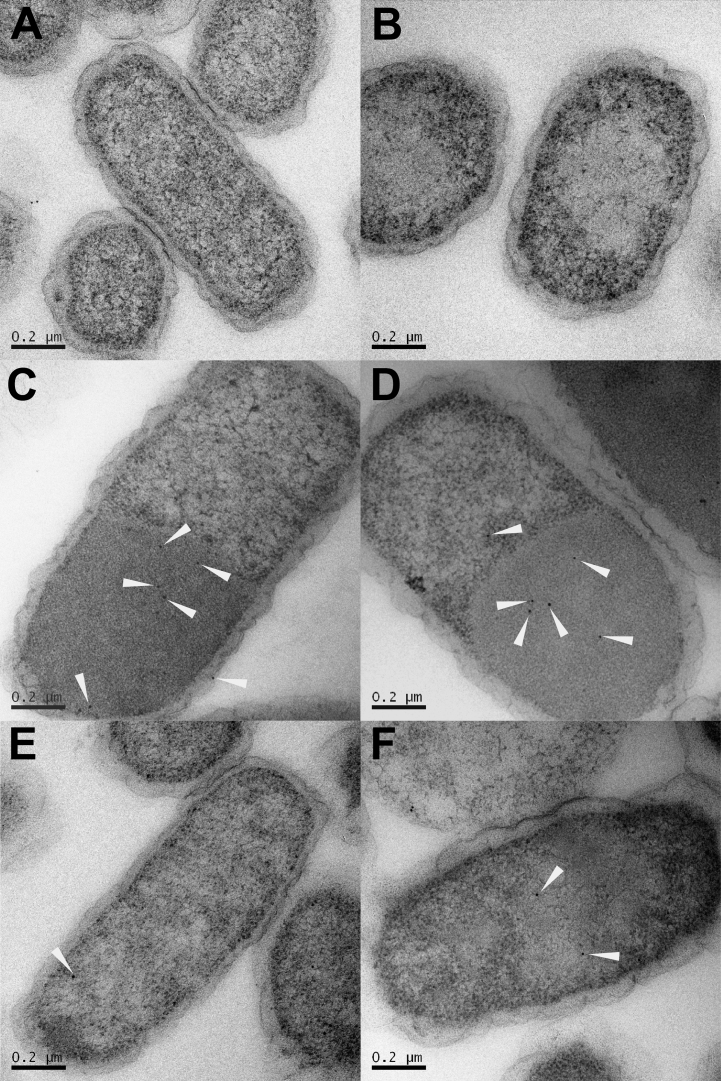
Thin sections of *E. coli* strains labelled with an anti-his antibody and then with a secondary antibody conjugated to 10 nm gold particles viewed under TEM (A) strain that lacks shell proteins and 1,2-propanediol producing enzymes (control strain) (B) strain producing shell proteins only (control strain) (C) 1,2-propanediol producing enzymes tagged with targeting peptides (D) 1,2-propanediol producing enzymes tagged with targeting peptides and shell proteins (E) untagged 1,2-propanediol producing enzymes (F) untagged 1,2-propanediol producing enzymes and shell proteins. White pointers indicate gold particles.

Control stains housing either empty vectors (pET14b+pLysS) or producing only the BMCs (pLysS-*pduABJKNU*) showed a small amount of antibody binding around the membranes of the cells (Fig. 6A and B); this is likely due to nonspecific binding. Aggregates, or large protein inclusions, are visible in approximately 100% of observed cells expressing the P18/D18-tagged proteins, regardless of whether BMCs are present. It is in these aggregate areas that the vast majority of antibody binding occurs (Fig. 6C and D). Such protein inclusions are not seen in cells expressing un-tagged enzymes (Fig. 6E and F), suggesting that it is the presence of the targeting peptides that facilitates the aggregation of proteins. In strains producing shell proteins, BMCs can be clearly seen grouped together within the cytoplasm of the cells when viewed by TEM (Supplementary Fig. S10). In the micrographs shown here the BMCs have low contrast because of the nature of sample preparation for immunolabelling (Fig. 6B, D and F). Only very few gold particles can be seen in the strain producing tagged enzymes and microcompartments in the region of the BMCs.

The combined data from both the 1,2-propanediol production studies and the TEM analysis of thin sections of bacteria from the various strains all suggest that maximum 1,2-propanediol production is due to the formation of a large active enzyme-inclusion body within the cell. The presence of the D18 and P18 fusion tags on the four enzymes (GldA, DhaK, MgsA and FucO) appears to result in the formation of a large protein aggregate when the four-tagged enzymes are co-produced. This large aggregate forms both in the presence and absence of BMCs.

## 4 Conclusions

Previously, we had shown that targeting of a two-enzyme heterologous pathway for ethanol production to engineered BMCs significantly increased the in vivo product yield in *E. coli.* Here, we have chosen to expand and apply our earlier findings to a pathway with industrial significance by engineering recombinant BMCs to house a four-enzyme pathway for the production of 1,2-propanediol from glycerol (GldA, DhaK, MgsA and FucO) in *E. coli*. Glycerol is a cheap and widely available source for the production of 1,2-propanediol, a valuable commodity chemical that is currently obtained from fossil fuel derivatives.

The first stage of the investigation into 1,2-propanediol synthesis in BMCs was to determine the effect of the BMC-targeting peptides, D18 and P18, on the pathway enzymes. The in vitro data presented in this study has revealed that the fusion of targeting peptides to the individual pathway enzymes has a variable effect on the properties of each protein. Differences can be seen in the levels of the protein, stability and in enzymatic activity. Three of the four tagged enzymes (GldA, MgsA and FucO) had lowered specific activities, caused by the addition of the 18 amino acid targeting sequences onto the N-terminus of the proteins. Moreover, the presence of the different peptide fusions, D18 and P18, had altered effects on the activity of the same enzyme. The solubility of each protein was also affected by the attachment of the peptide fusion, with GldA forming large inclusion bodies in the majority of cells observed by TEM when fused with a targeting peptide compared to un-tagged GldA.

To demonstrate that the D18 or P18 peptide was able to localise, individually, the 1,2-propanediol enzymes to the BMC, the four tagged enzymes were separately co-produced with the ‘empty’ Pdu BMC. In each case the tagged enzyme was found to co-purify with the BMC, suggesting that that the enzyme had been correctly targeted to the BMC. This in itself does not prove that the tagged enzyme has been encased within the recombinant BMC, merely that it associates with the BMC. Evidence that the D18 or P18 tagged proteins are internalised was provided by a protease protection assay using GFP as the cargo for the BMC. The presence of either the P18 or D18 peptide on the GFP protected the GFP from proteolysis but only when the tagged protein was co-produced with BMCs.

In an attempt to target the whole 1,2-propanediol pathway to a BMC, the genes encoding the most active of the individually tagged enzymes (P18-GldA, P18-DhaK, D18-MgsA and D18-FucO) were cloned consecutively on a single plasmid so that the whole pathway would be targeted to empty BMCs. In vivo analysis of 1,2-propanediol production of the strain showed that fusion of targeting peptides to all of the proteins involved in the synthesis of 1,2-propanediol resulted in an increase in product formation.

Rather unexpectedly, the presence of the microcompartment shell was not required for the increased product formation. In fact, the strain generating the most 1,2-propanediol produced tagged 1,2-propanediol pathway enzymes, but no shell proteins. This strain showed an increase in product formation of 245% OD-adjusted and 157% not OD-adjusted in comparison to the strain producing un-tagged enzymes; despite the lower in vitro activity of the individual tagged proteins compared to the un-tagged proteins. TEM analysis showed that co-production of all four proteins resulted in protein aggregation and deposition at the poles of nearly all cells and it is this aggregation that appears to provide a significant benefit to the efficiency of the pathway. Aggregation of our proteins of interest may be due to coiled coil interactions facilitated by the targeting peptides as previously shown for the P18 sequence, which appears to form a coiled coil dimer in solution (Lawrence et al., 2014). Pathway productivity could be further enhanced by introducing a cofactor-recycling enzyme; this has been previously implemented to enhance a related pathway for dihydroxyacetone production (Zhou et al., 2013). Through the use of a tightly regulated fermentation system it would reasonably be expected that pathway productivity could be further enhanced.

Hence, it could be assumed that the increased product yield is a result of concentrating enzymes into aggregates. As such this scaffold might result in increased channelling of substrates and products between proteins due to proximity effects. The formation of this aggregate may be a mimic of what happens in nature during BMC formation. For instance, in the case of the carboxysome, the cargo enzymes initially condense and are then encased by the shell of the BMC. The reason inclusion bodies are observed in this study even when shell proteins are co-produced is that the expression of the pathway genes is under the control of a strong T7 promoter which leads to disproportional ratios of cargo enzyme to shell proteins. Surplus protein, which is not encapsulated, is aggregated. In wild type operons gene expression and thus protein levels are controlled by regulatory elements, promoters and ribosome binding sites of various strengths.

In summary, the work described in this report demonstrates that the presence of short targeting peptides can not only convert individual fusion proteins but also whole pathways into active aggregates that allow for increased product yield in vivo. The aggregations of multiple enzymes allows for increased localised concentrations of enzymes and intermediates within the cell resulting in a higher product yield. In essence, these metabolic aggregates that are formed by the attachment of BMC-targeting peptides represent BMCs without shells. Hence, engineered protein aggregation for instance through the design of coiled coil interactions may hold an important role in the future of metabolic engineering for the production of commodity products. Active protein aggregates may have prolonged extracellular activity and therefore be more robust than BMCs. The use of BMCs for pathway localisation still remains desirable in the field of metabolic engineering. Pathways involved in the microbial production of biofuels often contain toxic intermediates and therefore compartmentalisation may reduce the negative effect of these toxic intermediates on the cell and thus increase fitness and productivity. Here we have shown that the formation of recombinant BMCs requires the coordinated production of protein cargo in a controlled manner for the construction of bioreactors of predictable functionality.

## Footnotes

Appendix A Supplementary data associated with this article can be found in the online version at doi:10.1016/j.ymben.2016.02.007.
