# Supplementary material for: Employing bacterial microcompartment technology to engineer a shell-free enzyme-aggregate for enhanced 1,2-propanediol production in Escherichia coli
Source: Metab Eng. 2016 Jul;36:48–56. doi: 10.1016/j.ymben.2016.02.007 (PMC4909751; doi:10.1016/j.ymben.2016.02.007)
Supplement: Supplementary file 1 — Supplementary material [file mmc1.docx]

**Supplementary information for:**

Employing bacterial microcompartment technology to engineer a shell-free enzyme-aggregate for enhanced 1,2-propanediol production in *Escherichia coli*

Matthew J. Lee^1^, Ian R. Brown^1^, Rokas Juodeikis^1^, Stefanie Frank^1^ and Martin J. Warren^1^,

^1^ School of Biosciences, University of Kent, Giles Lane, Canterbury, Kent, CT2 7NJ, U.K.

Corresponding authors:

Martin J. Warren,

Phone: 00 44 (0)1227 824690. E-mail: m.j.warren@kent.ac.uk.

School of Biosciences, University of Kent, Giles Lane, Canterbury, Kent, CT2 7NJ, U.K.

Stefanie Frank

Phone: 00 44 (0)1227 824692. E-mail: S.Frank@kent.ac.uk

School of Biosciences, University of Kent, Giles Lane, Canterbury, Kent, CT2 7NJ, U.K.

### Supplementary Table 1: Strains used in this study

| Strain | Genotype | Source |
| --- | --- | --- |
| JM109 | endA1, recA1, gyrA96, thi, hsdR17 (rk–, mk+), relA1, supE44, Δ(lac-proAB), [F′, traD36, proAB, laqIqZΔM15] | Promega |
| BL21 (DE3) | F– ompT hsdSB (rB– mB–) gal dcm (DE3) | Novagen |
| BL21 (DE3) pLysS | F- ompT hsdSB(rB- mB-) gal dcm (DE3) pLysS (CamR) | Novagen |

### Supplementary Table 2: Plasmids used in this study

| Plasmid name | Description | Source |
| --- | --- | --- |
| pET14b | Overexpression vector containing N-terminal hexahistidine-tag, modified to include an *Spe*I site 5’ of *Bam*HI | Novagen |
| pET14b-D18 | Overexpression vector containing an N-terminal D18 targeting tag followed by a short amino acid linker (AMGSS) then a hexahistidine-tag | This study |
| pET14b-P18 | Overexpression vector containing an N-terminal P18 targeting tag followed by a short amino acid linker (PMGSS) then a hexahistidine-tag | This study |
| pLysS | Basal expression suppressor | Novagen |
| pLysS-2 | Basal expression suppressor containing the T7 promoter-MCS-T7 terminator cassette from pET14b | Parsons *et al*., 2008 |
| pLysS-PduABJKNU | pLysS-2 containing genes required for the formation of empty BMCs | Parsons *et al*., 2010 |
| pET14b-gldA | PCR product of gldA ligated into *Nde*I/*Spe*I sites of pET14b | This study |
| pET14b-dhaK | PCR product of dhaK ligated into *Nde*I/*Spe*I sites of pET14b | This study |
| pET14b-mgsA | PCR product of mgsA ligated into *Nde*I/*Spe*I sites of pET14b | This study |
| pET14b-fucO | PCR product of fucO ligated into *Nde*I/*Spe*I sites of pET14b | This study |
| pET14b-GFP-SsrA | PCR product of gfp-ssrA ligated into *Nde*I/*Spe*I sites of pET14b | This study |
| pET14b-D18-gldA | *Nde*I/*Spe*I fragment of pET14b-gldA ligated into *Nde*I/*Spe*I sites of pET14b-D18 | This study |
| pET14b-D18-dhaK | *Nde*I/*Spe*I fragment of pET14b-dhaK ligated into *Nde*I/*Spe*I sites of pET14b-D18 | This study |
| pET14b-D18-mgsA | *Nde*I/*Spe*I fragment of pET14b-mgsA ligated into *Nde*I/*Spe*I sites of pET14b-D18 | This study |
| pET14b-D18-fucO | *Nde*I/*Spe*I fragment of pET14b-fucO ligated into *Nde*I/*Spe*I sites of pET14b-D18 | This study |
| pET14b-D18-GFP-SsrA | *Nde*I/*Spe*I fragment of pET14b-GFP-SsrA ligated into *Nde*I/*Spe*I sites of pET14b-D18 | This study |
| pET14b-P18-gldA | *Nde*I/*Spe*I fragment of pET14b-gldA ligated into *Nde*I/*Spe*I sites of pET14b-P18 | This study |
| pET14b-P18-dhaK | *Nde*I/*Spe*I fragment of pET14b-dhaK ligated into *Nde*I/*Spe*I sites of pET14b-P18 | This study |
| pET14b-P18-mgsA | *Nde*I/*Spe*I fragment of pET14b-mgsA ligated into *Nde*I/*Spe*I sites of pET14b-P18 | This study |
| pET14b-P18-fucO | *Nde*I/*Spe*I fragment of pET14b-fucO ligated into *Nde*I/*Spe*I sites of pET14b-P18 | This study |
| pET14b-P18-GFP-SsrA | *Nde*I/*Spe*I fragment of pET14b-GFP-SsrA ligated into *Nde*I/*Spe*I sites of pET14b-P18 | This study |
| pML-1 | *Xba*I/*Eco*RI fragment from pET14b-fucO ligated into *Xba*I/*Eco*RI sites of pET14b-gldA | This study |
| pML-2 | *Xba*I/*Eco*RI fragment from pET14b-D18-fucO ligated into *Spe*I/*Eco*RI sites of pET14b-P18-gldA | This study |
| pML-3 | *Xba*I/*Hind*III fragment from pET14b-mgsA ligated into *Spe*I/*Hind*III sites of pET14b-dhaK | This study |
| pML-4 | *Xba*I/*Hind*III fragment from pET14b-D18-mgsA ligated into *Spe*I/*Hind*III sites of pET14b-P18-dhaK | This study |
| pML-5 | *Xba*I/*Cla*I fragment from pML-3 ligated into *Spe*I/*Cla*I sites of pML-1 | This study |
| pML-6 | *Xba*I/*Cla*I fragment from pML-4 ligated into SpeI/ClaI sites of pML-2 | This study |

**Supplementary Table 3:** Oligonucleotides used in this study, restriction sites are underlined

| Name | Sequence 5’ – 3’ |
| --- | --- |
| GldA_NdeI_FW | CATCATATGGACCGCATTATTCAATCACC |
| GldA_SpeI_RV | CATACTAGTTTATTCCCACTCTTGCAGG |
| dhaK_NdeI_FW | CGCCATATGTCTCAATTCTTTTTTAACCAACGCACC |
| dhaK_SpeI_RV | CATACTAGTTTAGCCCAGCTCACTCTCCGC |
| mgsA_NdeI_FW | CATCATATGGAACTGACGACTCGCACTTTACC |
| mgsA_SpeI_RV | CATACTAGTTTACTTCAGACGGTCCGCGAG |
| fucO_NdeI_FW | CCGCATATGGCTAACAGAATGATTCTG |
| fucO_SpeI_RV | CCTACTAGTTTACCAGGCGGTATGG |
| GFP_NdeI_FW | GTACATATGAGCAAAGGAGAAGAACTTTTC |
| GFP-SsrA_SpeI_RV | GACACTAGTTTAAGCTGCTAAAGCGTAGTTTTCGTCGTTTGCTGCTTTGTACAGCTCATCCATGCC |

**
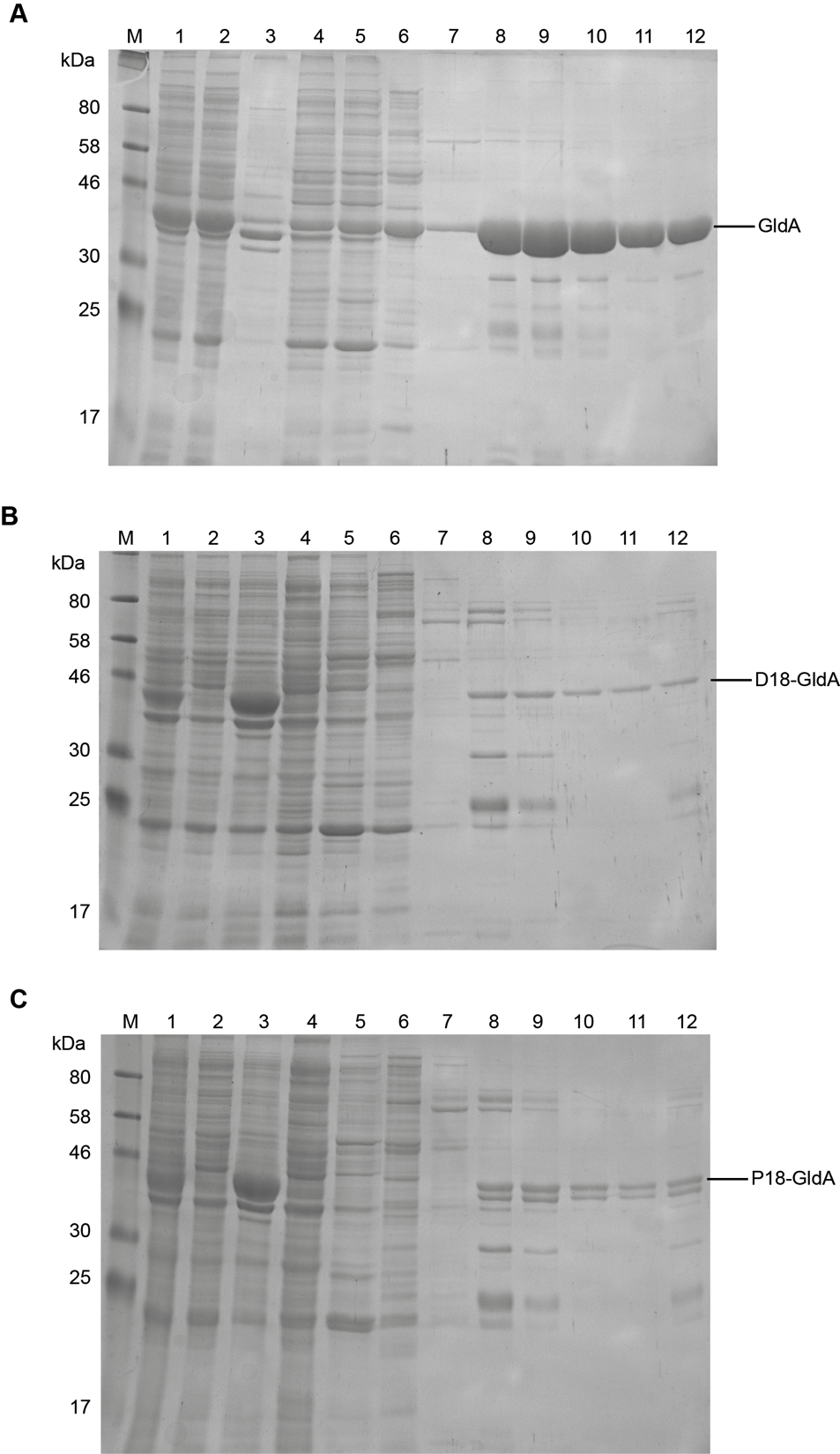
**

**Supplementary Figure S1:** SDS-PAGE of GldA purified by IMAC (a) GldA (b) D18-GldA (c) P18-GldA; In comparison to a molecular weight marker. Lane 1 – lysate (2 μl), lane 2 – supernatant after centrifugation (2 μl), lane 3 – pellet after centrifugation (10 μl), lane 4 – supernatant flow through (8 μl), lane 5 – binding buffer flow through (5 mM imidazole) (10 μl), lane 6 – wash buffer 1 flow through (50 mM imidazole) (10 μl), lane 7 – wash buffer 2 flow through (100 mM imidazole) (10 μl), lanes 8– 11 – elution fractions 4-7 (400 mM imidazole) (5 μl), lane 12 – sample after buffer exchange (5 μl).

**
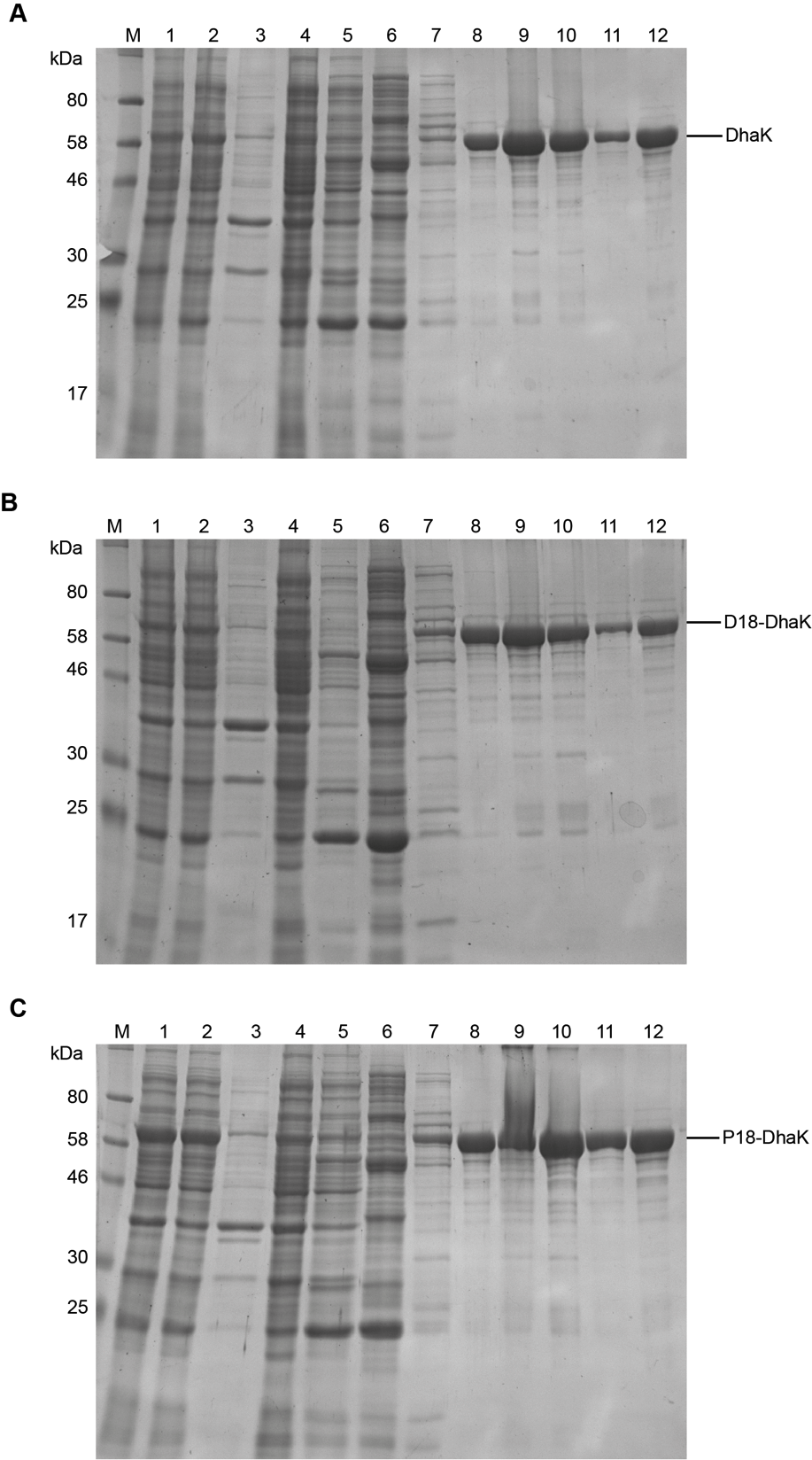
**
**Supplementary Figure S2:** SDS-PAGE of DhaK purified by IMAC (a) DhaK (b) D18-DhaK (c) P18-DhaK; In comparison to a molecular weight marker. Lane 1 – lysate (3 μl), lane 2 – supernatant after centrifugation (3 μl), lane 3 – pellet after centrifugation (10 μl), lane 4 – supernatant flow through (10 μl), lane 5 – binding buffer flow through (5 mM imidazole) (10 μl), lane 6 – wash buffer 1 flow through (50 mM imidazole) (10 μl), lane 7 – wash buffer 2 flow through (100 mM imidazole) (10 μl), lanes 8– 11 – elution fractions 2-5 (400 mM imidazole) (3 μl), lane 12 – sample after buffer exchange (3 μl).

**
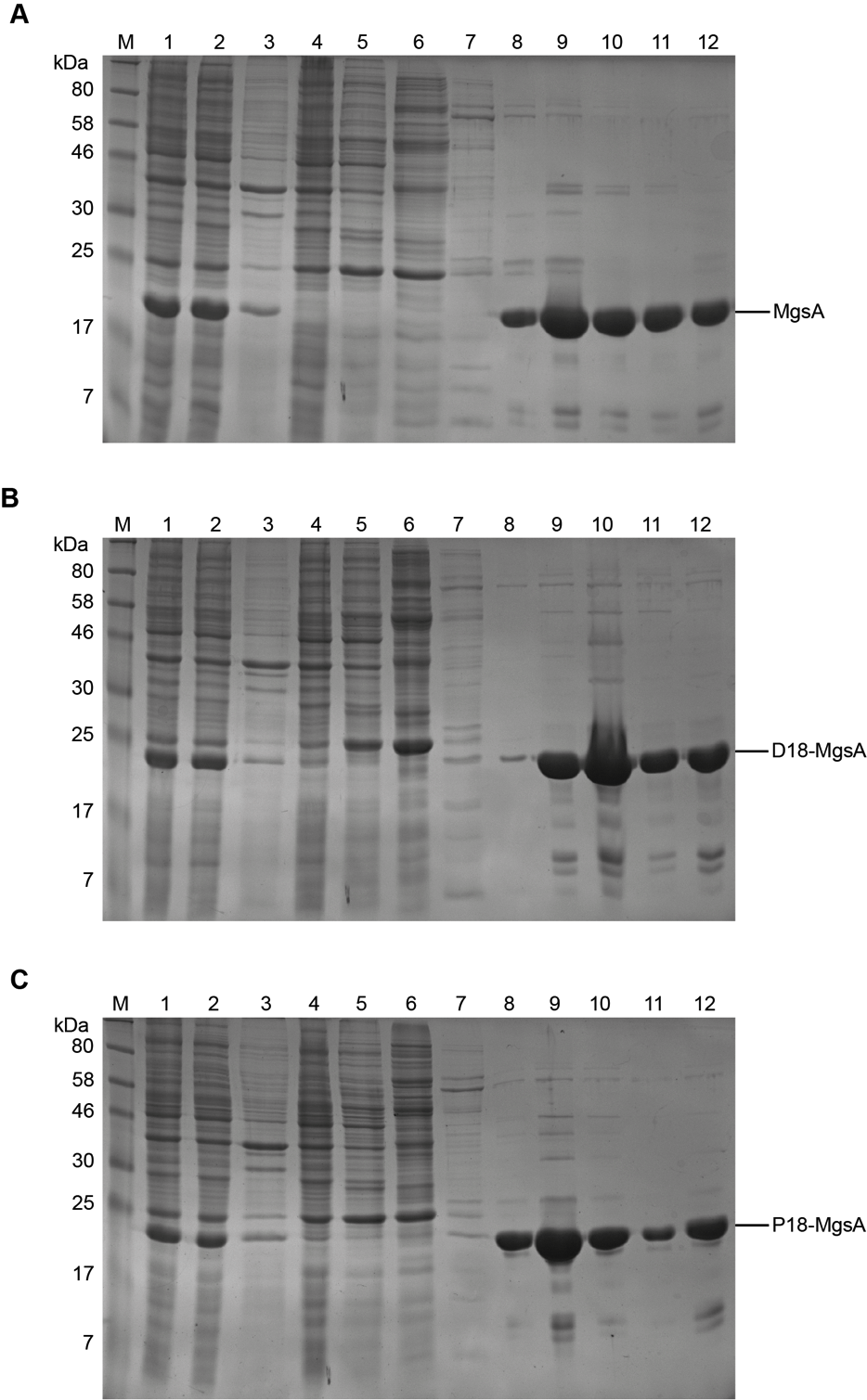
**

**Supplementary Figure S3:** SDS-PAGE of MgsA purified by IMAC. (a) MgsA (b) D18-MgsA (c) P18-MgsA; In comparison to a molecular weight marker. Lane 1 – lysate (2 μl), lane 2 – supernatant after centrifugation (2 μl), lane 3 – pellet after centrifugation (10 μl), lane 4 – supernatant flow through (3 μl), lane 5 – binding buffer flow through (5 mM imidazole) (10 μl), lane 6 – wash buffer 1 flow through (50 mM imidazole) (10 μl), lane 7 – wash buffer 2 flow through (50 mM imidazole) (10 μl), lanes 8– 11 – elution fractions 2-5 (400 mM imidazole) (2 μl), lane 12 – sample after buffer exchange (2 μl).

**
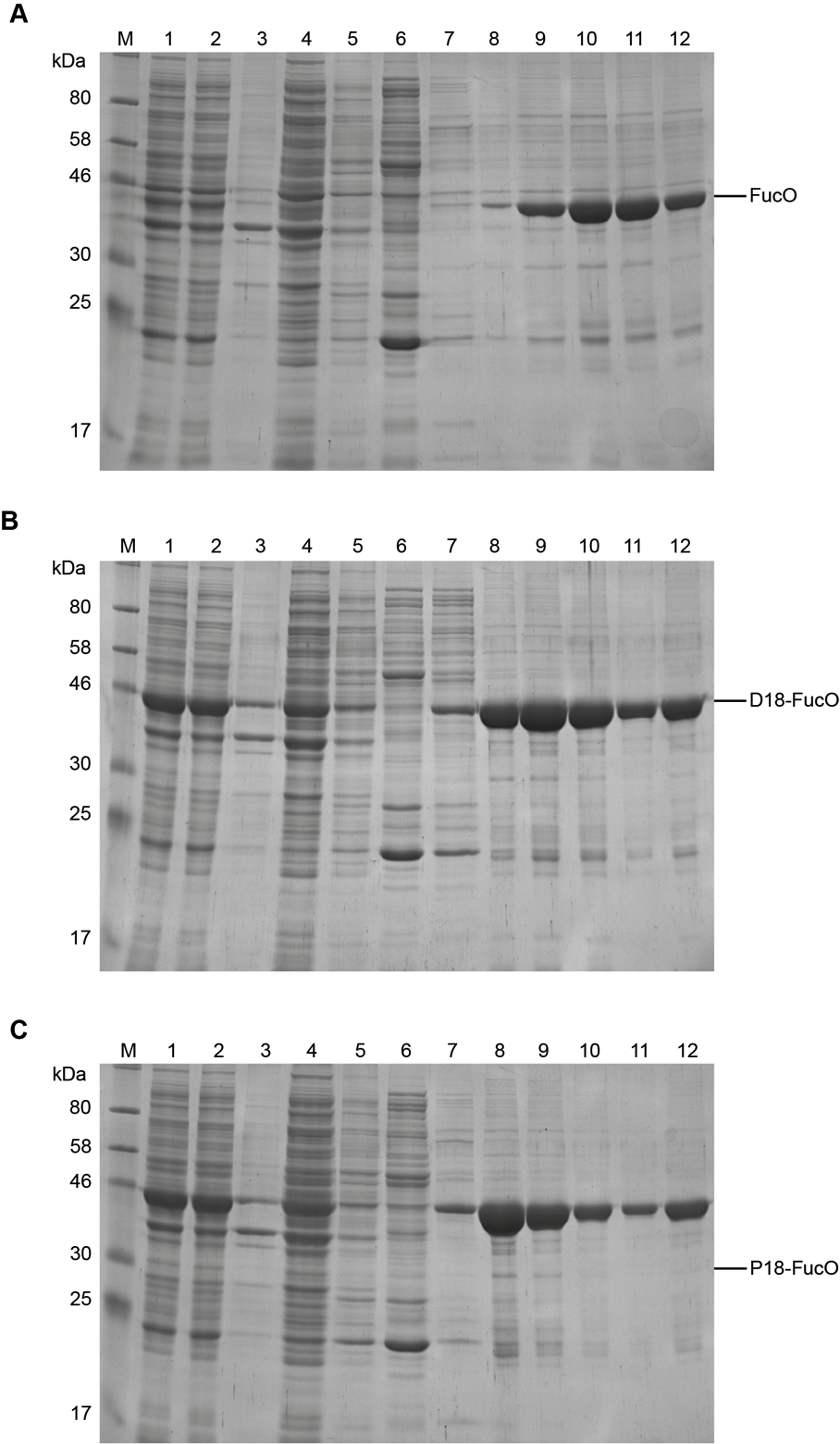
**

**Supplementary Figure S4:** SDS-PAGE of FucO purified by IMAC (a) FucO (b) D18-FucO (c) P18-FucO; In comparison to a molecular weight marker. Lane 1 – lysate (4 μl), lane 2 – supernatant after centrifugation (4 μl), lane 3 – pellet after centrifugation (10 μl), lane 4 – supernatant flow through (10 μl), lane 5 – binding buffer flow through (5 mM imidazole) (10 μl), lane 6 – wash buffer 1 flow through (50 mM imidazole) (10 μl), lane 7 – wash buffer 2 flow through (100 mM imidazole) (10 μl), lanes 8– 11 – elution fractions 3-6 (400 mM imidazole) (2 μl), lane 12 – sample after buffer exchange (2 μl).


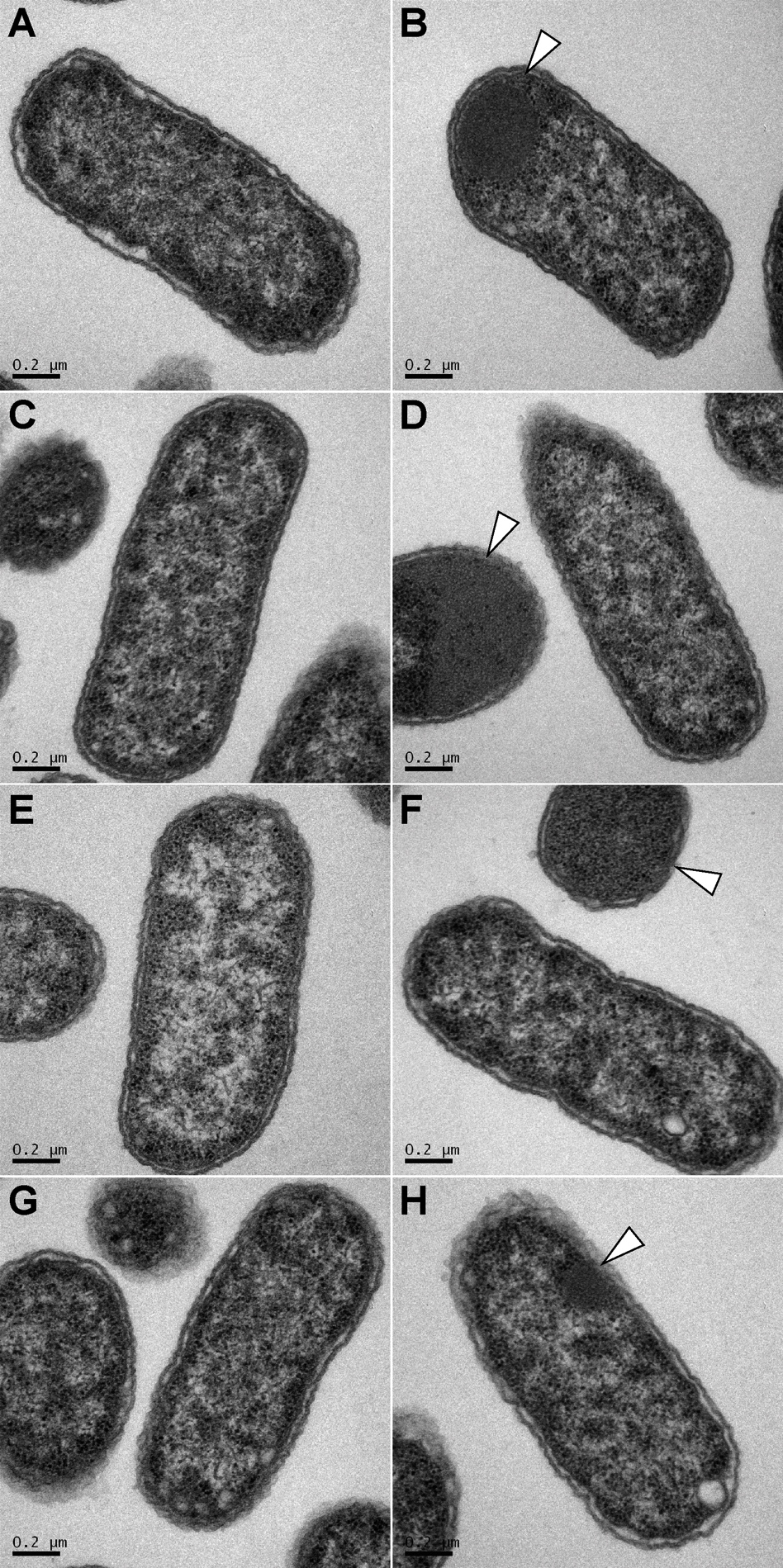

**Supplementary Figure S5:** TEM analysis of strains expressing (A) GldA (B) P18- GldA (C) DhaK (D) P18-DhaK (E) MgsA (F) D18-MgsA (G) FucO (H) D18-FucO. Arrows indicate protein aggregates. Scale bar shows 0.2 μm


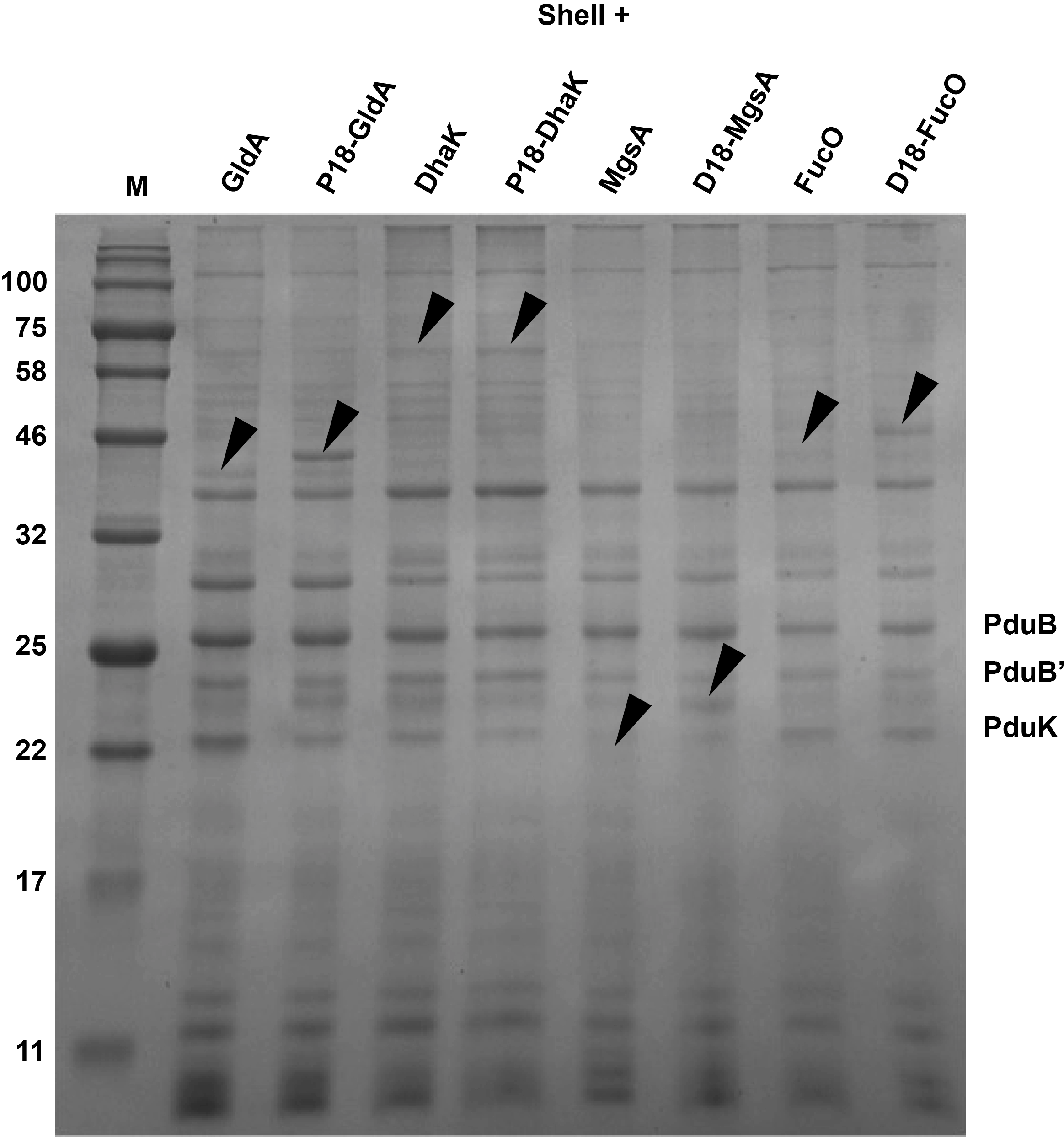

**Supplementary Figure S6** SDS-PAGE analysis of purified BMCs co-produced with tagged or untagged GldA, DhaK, MgsA or FucO.

**
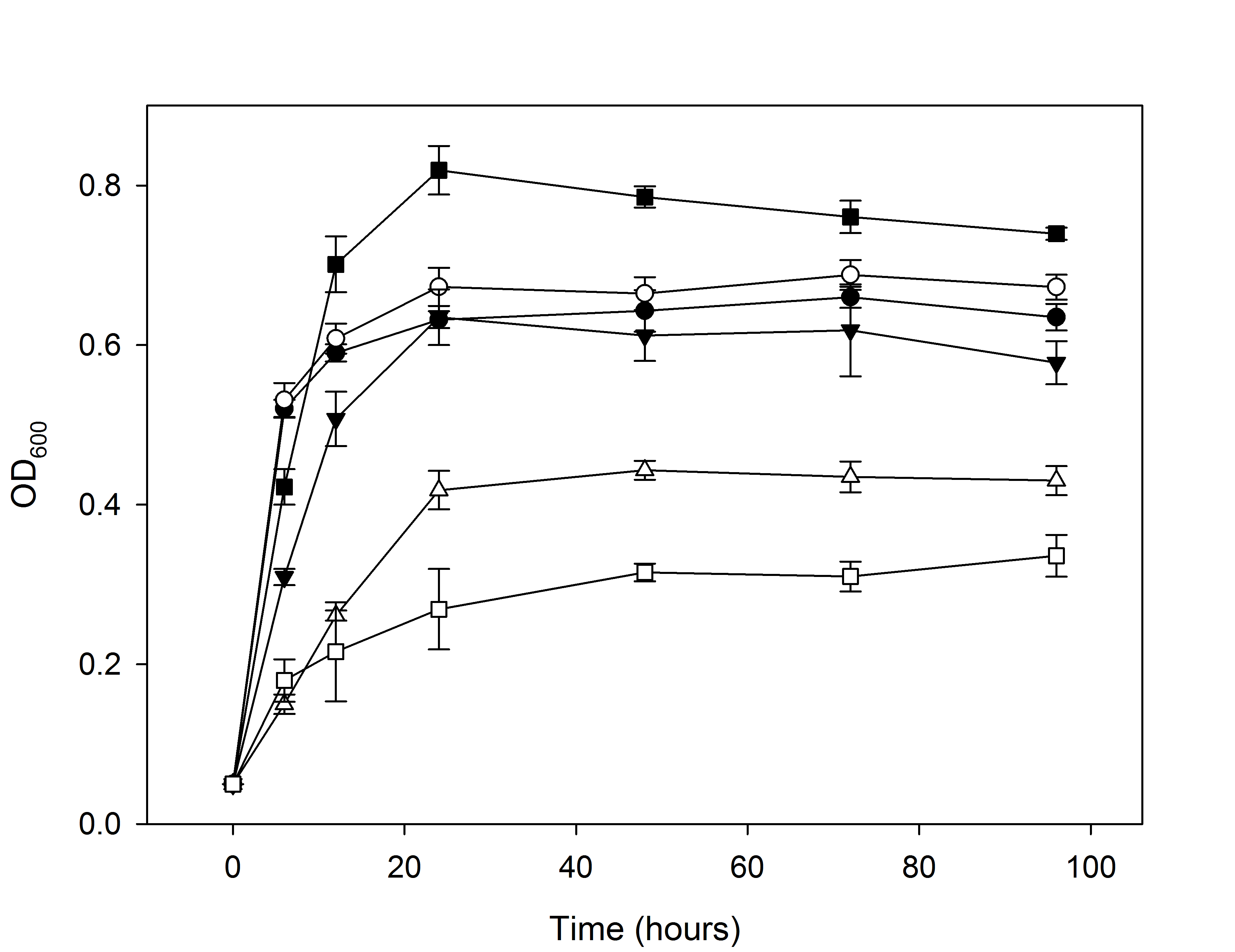
**
**Supplementary Figure S7:** Growth curves of strains producing 1,2-propanediol and control strains shown as OD_600_ against time (hours). *E. coli* strain that lacks shell proteins and 1,2-propanediol producing enzymes (control strain) (●), Shell proteins only (control strain) (○), untagged 1,2-propanediol producing enzymes (▼), 1,2-propanediol producing enzymes tagged with targeting peptides (△), untagged 1,2-propanediol producing enzymes and shell proteins (■),1,2-propanediol producing enzymes tagged with targeting peptides and shell proteins (□). Data points represent an average of three independent experiments; standard deviations are represented by error bars.


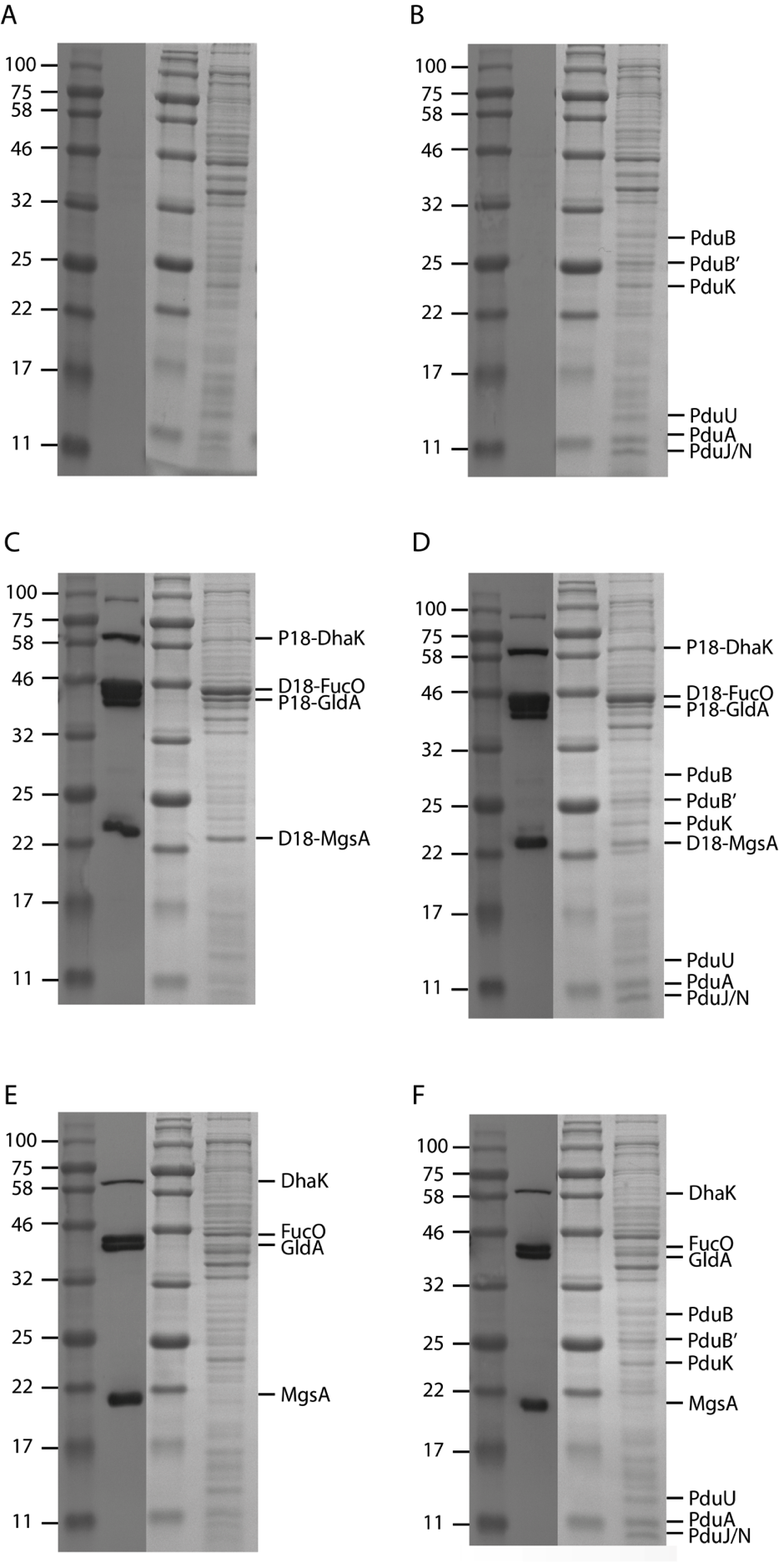
 **Supplementary Figure S8:** SDS-PAGE and western blot analysis of final whole cell samples of (A) strain that lacks shell proteins and 1,2-propanediol producing enzymes (control strain) (B) shell proteins only (PduABB’JKNU) (control strain) (C) P18/D18-tagged-DhaK, MgsA, GldA and FucO (D) P18/D18-tagged-DhaK, MgsA, GldA and FucO + PduABB’JKNU (E) untagged-DhaK, MgsA, GldA and FucO (F) untagged-DhaK, MgsA, GldA and FucO + PduABB’JKNU adjusted to the same OD_600_ (2.5)


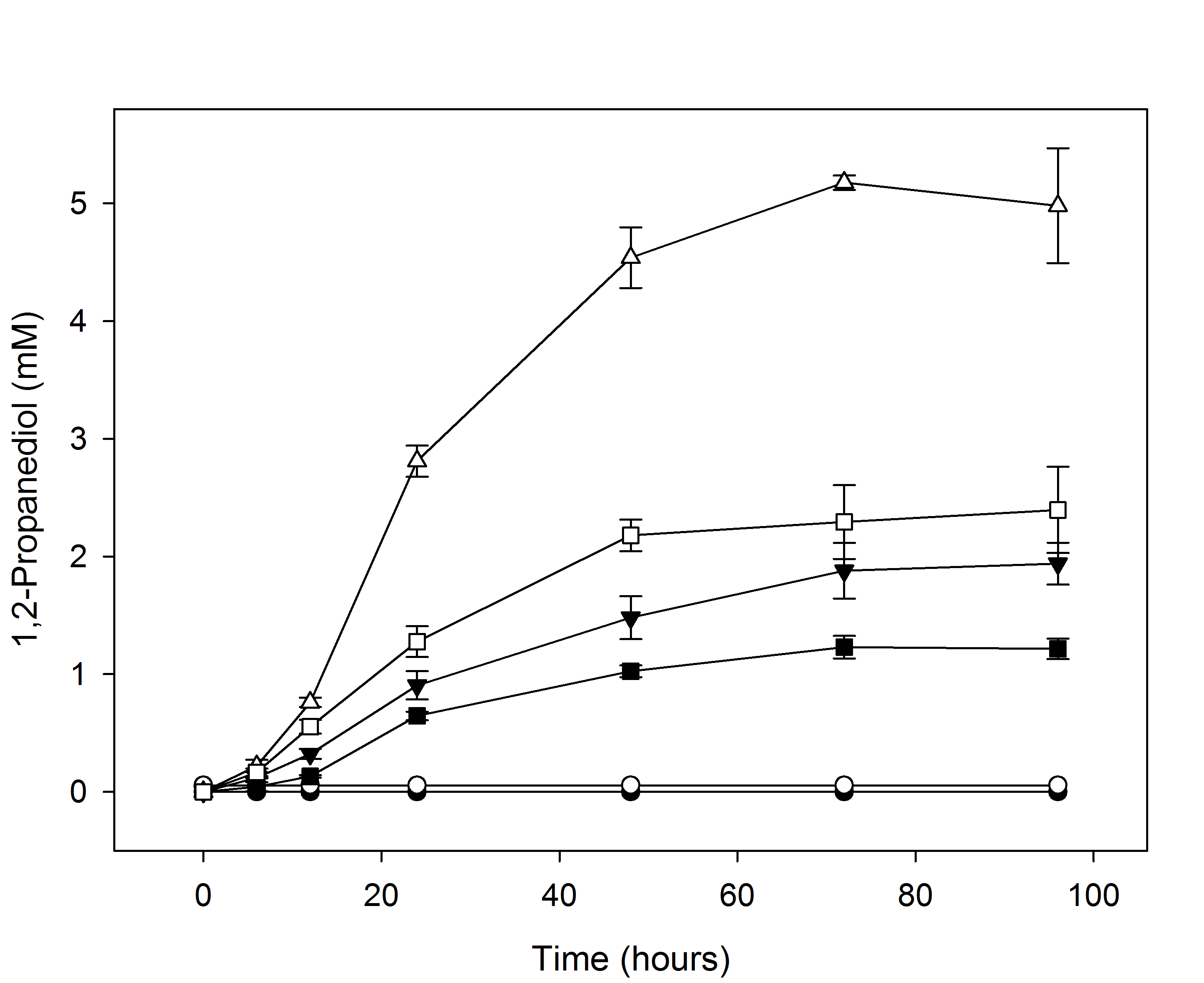


**Supplementary Figure S9** *In vivo* 1,2-propanediol production. The graph shows the 1,2-propanediol content over 96 h in the growth medium in of strains that lack shell proteins and 1,2-propanediol producing enzymes (control) (●), shell proteins only (control strain) (○), untagged 1,2-propanediol producing enzymes (▼), 1,2-propanediol producing enzymes tagged with targeting peptides (△),untagged 1,2-propanediol producing enzymes and shell proteins (■),1,2-propanediol producing enzymes tagged with targeting peptides and shell proteins (□). Data points represent an average of three independent experiments; standard deviations are represented by error bars.


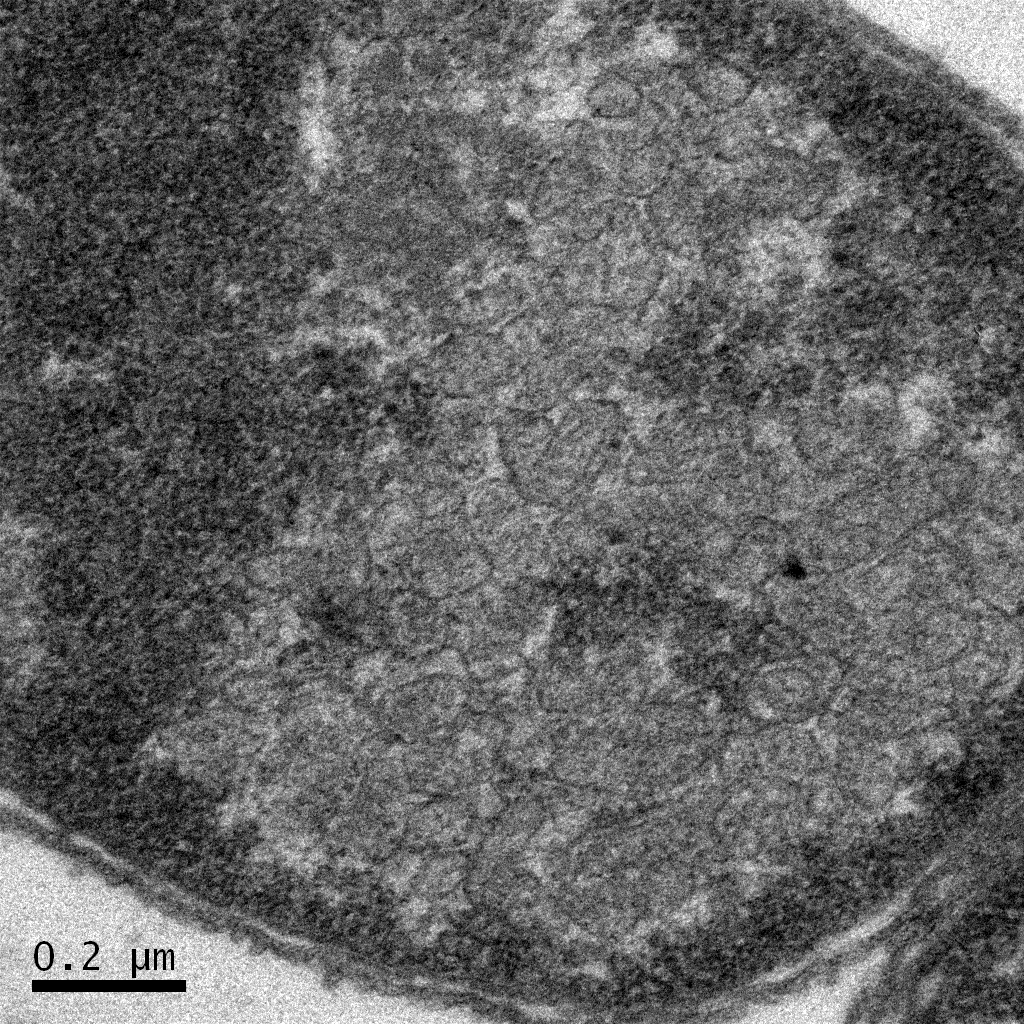

**Supplementary Figure S10:** TEM micrograph showing thin section of *E. coli* strain BL21*(DE3) transformed with pLysS-PduABJKNU.

Supplementary methods

Embedding of strains for TEM analysis

50 ml of LB was inoculated with one colony and grown at 37 °C with shaking to an OD600 of ~ 0.4, cells were harvested by centrifugation at 3000 x g for 10 minutes. The cell pellet was resuspended in 2 ml 2.5% Glutaraldehyde in 100 mM sodium cacodylate buffer pH 7.2 (CAB) and fixed for 2 hours with gentle rotating (20 rpm). Cells were pelleted by centrifugation at 6000 x g for 2 minutes and were washed twice for 10 minutes with 100 mM CAB. Cells were post-fixed with 1% osmium tetroxide in 100 mM CAB for 2 hours and subsequently washed twice with dH_2_O. Cells were dehydrated by incubation in an ethanol gradient, 50% EtOH for 10 minutes, 70% EtOH overnight, 90% EtOH for 10 minutes followed by three 10 minute washes in 100% dry EtOH. Cells were then washed twice with propylene oxide for 15 minutes. Cell pellets were embedded by resuspension in 1 ml of a 1:1 mix of propylene oxide and Agar LV Resin and incubated for 30 minutes with rotation. Cell pellets were infiltrated twice in 100% Agar LV resin. The cell pellet was re-suspended in fresh resin and transferred to a 1ml Beem embedding capsule, centrifuged for 5 minutes at 3000 x g to concentrate the cells to the tip of the mould and incubated for 20 hours at 60 °C to polymerise.

Sectioning and visualisation of samples

Samples were ultra-thin sectioned on a RMC MT-XL ultra-microtome with a diamond knife (diatome 45°) sections (60 – 70 nm) were collected on un-coated 300 mesh copper grids. Grids were stained by incubation in 4.5% uranyl acetate in 1% acetic acid solution for 45 minutes followed by washing in a stream of dH_2_O. Grids were then stained with Reynolds lead citrate for 7 minutes followed by washing in a stream of dH_2_O

Electron microscopy was performed using a JEOL-1230 transmission electron microscope equipped with a Gatan multiscan digital camera operated at an accelerating voltage of 80 kV

Supplementary references

Parsons, J.B., Dinesh, S.D., Deery, E., Leech, H.K., Brindley, A.A., Heldt, D., Frank, S., Smales, C.M., Lünsdorf, H., Rambach, A., Gass, M.H., Bleloch, A., McClean, K.J., Munro, A.W., Rigby, S.E., Warren, M.J., Prentice, M.B. (2008) Biochemical and structural insights into bacterial organelle form and biogenesis. J Biol Chem. 283:14366-14375.

Parsons, J.B., Frank, S., Bhella, D., Liang, M., Prentice, M.B., Mulvihill, D.P., Warren, M.J. (2010) Synthesis of empty bacterial microcompartments, directed organelle protein incorporation, and evidence of filament-associated organelle movement. Mol Cell. **38**:305-315.
